# Building awareness and capacity of bioinformatics and open science skills in Kenya: a sensitize, train, hack, and collaborate model

**DOI:** 10.3389/frma.2023.1070390

**Published:** 2023-05-30

**Authors:** Pauline Karega, David K. Mwaura, Kennedy W. Mwangi, Margaret Wanjiku, Michael Landi, Caleb K. Kibet

**Affiliations:** ^1^International Center of Insect Physiology and Ecology, Nairobi, Kenya; ^2^Department of Biochemistry, University of Nairobi, Nairobi, Kenya; ^3^Institute of Primate Research (IPR), Nairobi, Kenya; ^4^International Livestock Research Institute (ILRI), Nairobi, Kenya; ^5^Department of Biology, San Diego State University, San Diego, CA, United States; ^6^Department of Bioinformatics, Swedish University of Agricultural Sciences, Uppsala, Sweden; ^7^International Institute of Tropical Agriculture, Nairobi, Kenya

**Keywords:** open science, bioinformatics, BHKi, OpenScienceKE, Africa

## Abstract

We have applied the sensitize-train-hack-community model to build awareness of and capacity in bioinformatics in Kenya. Open science is the practice of science openly and collaboratively, with tools, techniques, and data freely shared to facilitate reuse and collaboration. Open science is not a mandatory curriculum course in schools, whereas bioinformatics is relatively new in some African regions. Open science tools can significantly enhance bioinformatics, leading to increased reproducibility. However, open science and bioinformatics skills, especially blended, are still lacking among students and researchers in resource-constrained regions. We note the need to be aware of the power of open science among the bioinformatics community and a clear strategy to learn bioinformatics and open science skills for use in research. Using the OpenScienceKE framework—Sensitize, Train, Hack, Collaborate/Community—the BOSS (Bioinformatics and Open Science Skills) virtual events built awareness and empowered researchers with the skills and tools in open science and bioinformatics. Sensitization was achieved through a symposium, training through a workshop and train-the-trainer program, hack through mini-projects, community through conferences, and continuous meet-ups. In this paper, we discuss how we applied the framework during the BOSS events and highlight lessons learnt in planning and executing the events and their impact on the outcome of each phase. We evaluate the impact of the events through anonymous surveys. We show that sensitizing and empowering researchers with the skills works best when the participants apply the skills to real-world problems: project-based learning. Furthermore, we have demonstrated how to implement virtual events in resource-constrained settings by providing Internet and equipment support to participants, thus improving accessibility and diversity.

## Introduction

Bioinformatics is the field that uses computational tools to capture, analyze, and interpret biological data (Bayat, 2002). Genome projects have increased manifold since the advent of cheaper next-generation sequencing technologies (Batley and Edwards, 2009), leading to an explosion of genomic sequences in public and private databases that need analysis and interpretation (Schneider et al., 2010). This explosion of data has also resulted in new analysis techniques and bioinformatics solutions to process them. Therefore, a skilled bioinformatics workforce is constantly required (Braga et al., 2021). The workforce would consist of researchers with the skills or core competencies listed by the International Society of Computational Biology (ISCB) (Mulder et al., 2018). Bioinformatics training in Kenya and most parts of the world occurs primarily in graduate programs and, in some cases, at the undergraduate level (Sayres et al., 2018). However, the graduate programs are usually tailored for those who want to specialize in bioinformatics and may not be ideal for those who want to use bioinformatics as a tool (Aron et al., 2021; Ras et al., 2021). Therefore, short training for skill development programs is essential to equip students with the necessary bioinformatics skills to conduct research and data analysis. Short training courses tend to have customized specificities not covered by traditional courses (Braga et al., 2021) and also have leeway in their design, factoring in a specific audience in attendance, a luxury that traditional courses do not have.

Data availability for reuse by researchers, especially from resource-constrained settings, results from a strong move toward open data sharing, a practice known as open science.

Open science is an umbrella term comprising open access to publications, open research data, open-source software, open collaboration, peer review, notebooks, educational resources, monographs, citizen science, or research crowdfunding. Data is only reusable when FAIR—Findable, Accessible, Interoperable, Reusable. Therefore, data-generating researchers must be trained to make their data FAIR. Open science skills are essential in bioinformatics to facilitate open, reproducible, and collaborative research stages in the research life cycle (OECD, 2015). Open science training is usually through informal training by grassroots communities through workshops, short courses, and MOOCs on open science. However, it is necessary to combine bioinformatics and open science training, which are traditionally not taught together in formal curricula in Kenya and surrounding countries (Mwangi et al., 2021) to facilitate the effective adoption of open practices in bioinformatics research.

The COVID-19 pandemic demonstrated the need for open science adoption in biomedical research (Okafor et al., 2022). Practices such as open data, open access, open source, and open peer review enabled quick and timely responses from researchers globally who were experiencing restrictive movements to work together collaboratively (Besançon et al., 2021). The demand further demonstrates the need for awareness of open science, more so, open science by design, where there is the intent to conduct research transparently and openly at all stages of the research life cycle. We note that in computational analysis, the scholarly contribution is the data and the code that generated the results, with a strong move in the field toward collaborative and reproducible research. Therefore, this study sought to empower researchers with skills and tools in bioinformatics through a series of virtual events dubbed Bioinformatics and Open Science Skills (BOSS) using the sensitize-train-hack-collaborate model. The main objective of the series of events was to blend open science and bioinformatics and to train researchers to conduct open, reproducible, and collaborative research in bioinformatics. This paper describes how we applied the model virtually and highlights the successes and challenges of organizing such events in a resource-constrained region.

## The BOSS events

**Figure 1:**
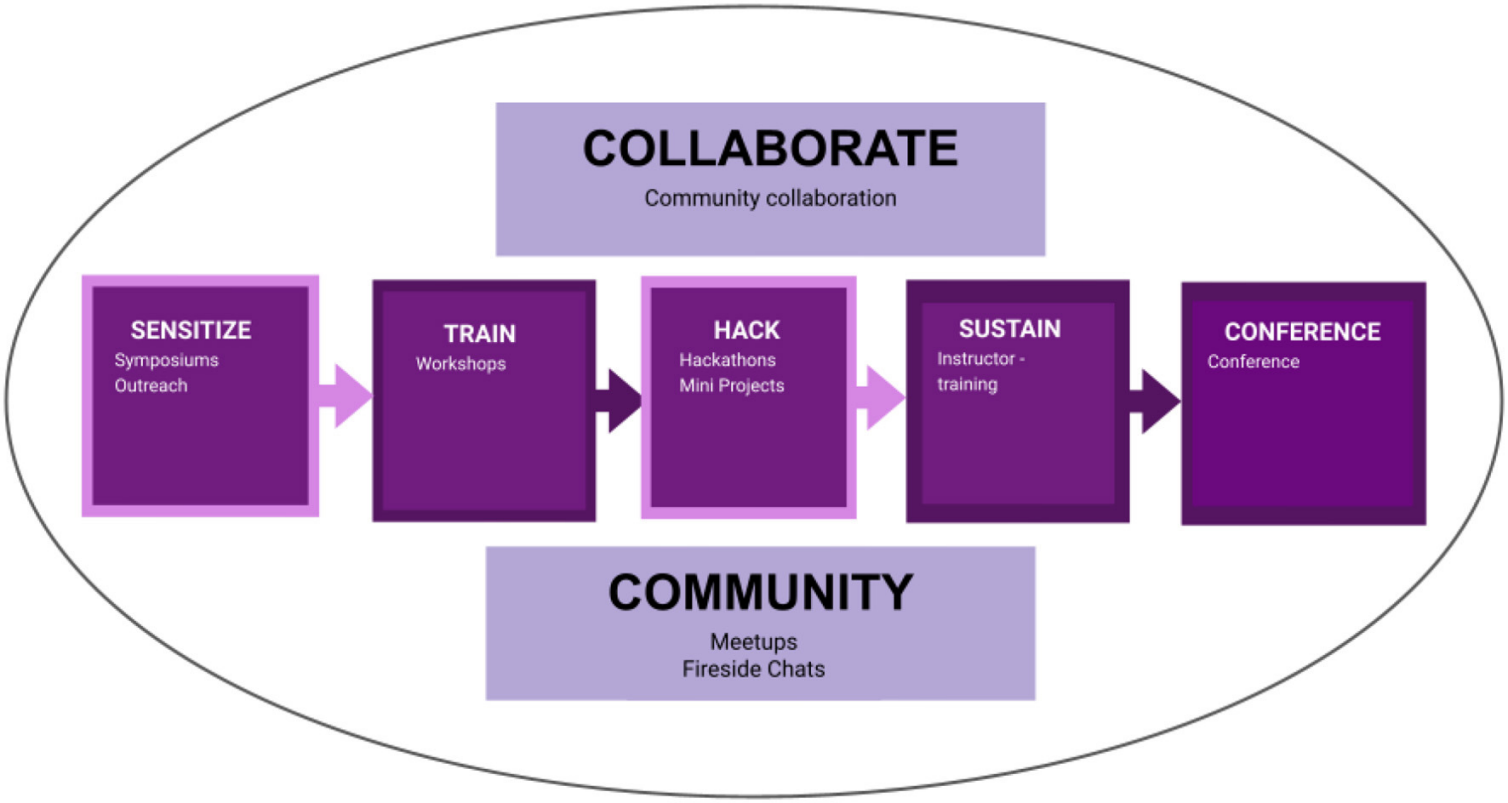
The model was adapted from the OpenScienceKE framework and used during the BOSS events. The sensitization phase involved the Open Science FAIR symposium; the training phase involved two parts; the BOSS workshop and Instructor training, the hack phase was done through mini-projects, which also encompassed the aspect of collaboration, and the conference covered the community aspect.

The BOSS events had five phases: sensitize through a symposium, training through workshops, hacking through mini-projects, sustain through instructor training, and a conference to showcase work done during the events and network. The main objective of the series of events was to blend open science and bioinformatics and to train researchers to conduct open, reproducible, and collaborative research in bioinformatics. We aimed to reach an audience with beginner, intermediate, and advanced knowledge of bioinformatics and open science. We adopted the sensitize-train-hack-collaborate model previously developed to empower researchers with open science skills (Mwangi et al., 2021). We adjusted the model to include sustainability, an opportunity to network and share work through conferences, and redefining collaboration and community to encompass all aspects of the model (Figure 1). The model and steps incorporated Bloom's taxonomy of effective learning to ensure that the content equipped trainees with knowledge and skills (Bloom, 1969). All the events were virtual using Zoom video conferencing.

### Sensitize: the open science FAIR symposium

Creating awareness and providing information on the need and benefits of practicing open science principles in bioinformatics formed the foundation step of the framework during its conception. Therefore, the FAIR Open Science Symposium aimed to create awareness of the need and the benefits of practicing open science in research (Table 1).

**Table 1:** The topics covered during the 5-day symposium.

**Day**	**Theme**
Day 1: 11 October 2021	Open Science
Day 2: 12 October 2021	Research data management
Day 3: 13 October 2021	Project Planning
Day 4: 14 October 2021	Reproducibility in research
Day 5: 15 October 2021	Contribution to open source projects: a practical approach

**Figure 2:**
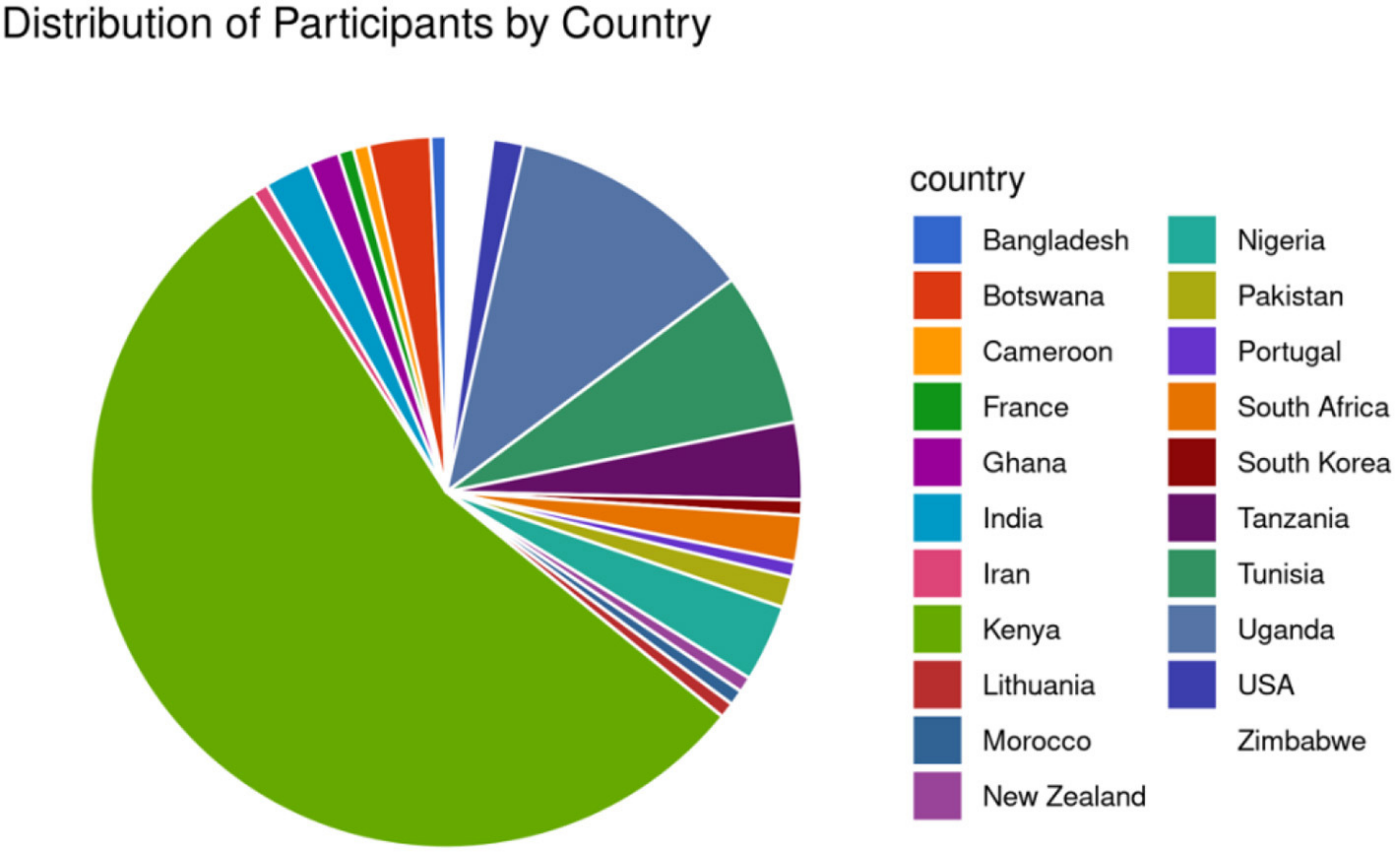
The proportion of participants registered for the Open Science FAIR symposium.

The symposium received 130 applications from various African countries such as Kenya, Uganda, Cameroon, Morocco, Nigeria, Tunisia, Botswana, Ghana, South Africa, Tanzania, and Zimbabwe (Figure 2). The event included participants outside Africa: the United States of America, India, Lithuania, France, Bangladesh, Iran, New Zealand, South Korea and Portugal. However, only 55 participants attended, with numbers varying throughout the week, with a minimum of 35. The active participants were mainly from Kenya, Uganda, Tanzania, the United States, Zimbabwe, South Africa, and South Korea.

The 5-day event had sessions that were in the form of talks, open panel discussions, and practical lessons. The sessions included expert presentations followed by panel discussions guided by questions from the audience. All sessions were accessible to all registered participants.

The open science session addressed the topics of the status of open science in Kenya and Africa, policies in higher education and research institutions, and the challenges faced, especially in the practice of open science. The speakers gave visual presentations on adopting open-access in Kenya and Africa, pointing out the growth of open access journals in the region.

The sessions on research data management aimed to sensitize the FAIR principles, introducing the participants to the definitions of each component of FAIR. The speakers covered scientific research data management, the importance of proper metadata handling, open data tools such as Dryad, persistent identifiers to monitor the use of open data and the need for clear research data management policies. A panel discussion of the day addressed issues such as confidence in sharing data openly due to fear of scooping, a significant hindrance to open science adoption in the Global South. The challenges noted in openly sharing data include ownership issues and misinterpretation of the data.

The project planning session targeted MSc and Ph.D. students, and researchers. The central theme of the session was “open by design research to foster reproducibility and collaboration.” These sessions motivated the benefits of reproducible research in promoting collaboration, avoiding misinformation, and ensuring continuity in research. The sessions also provided a foundation to open science through project planning. They highlighted the vast array of open science tools researchers could use in each step of the research life cycle. Amazon Cloud, Jupyter Notebooks, RStudio, Singularity and Docker Containers, Nextflow and Snakemake workflows, Dryad, and Figshare. The symposium also included a practical session to demonstrate how to contribute to open source projects using GitHub to give them an appreciation of the importance of open source in research.

Feedback from the participants after the sessions revealed that the symposium met their expectations, with the discussion on low-cost publication options for the Global South and the research findings and work done to map how open science is progressing in Kenya being a favorite. However, some noted the need for more practical sessions and discussions on open policy.

### Train phase: bioinformatics workshops

**Figure 3:**
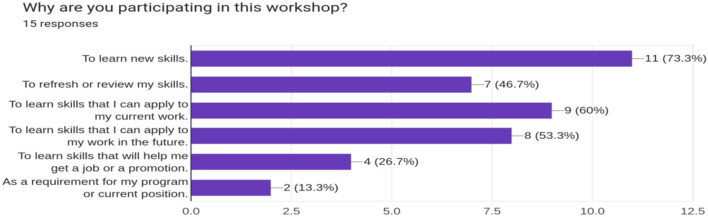
The response of the participants to their motivation to attend the bioinformatics workshops.

With participants aware of the need for open science in collaborative and reproducible bioinformatics research, the second phase aimed to empower participants by equipping them with bioinformatics and open science tools introduced in the symposium. BOSS events implemented this phase through a workshop that taught introductory bioinformatics and open science skills. The pre-workshop survey revealed that most trainees were interested in learning new skills to apply to their current and future work (Figure 3).

Of 68 applications, 35 undergraduate and 25 master's, and 7 Ph.D. students. We tailored the workshop content to target audiences with beginner to intermediate skills (Table 2).

**Table 2:** The training curriculum for the Bioinformatics and Open Science workshops.

**Day**	**Morning session**	**Time**	**Afternoon session (1400-1630hrs)**
Monday	Intro to sequencing technologies	9:00–10:00 am	•Introduction to High Performance Computing (HPC) •Assignments on data file formats and Introduction to Unix
	Data file formats	10:15–11:00 am	
	Introduction to unix	11:15–12:30 pm	
Tuesday	Advanced Linux, Awk, and Sed	9:00–11:00 pm	Assignments—Unix, Sed, and Awk
Wednesday	Quality control and assessment	9:00–10:00 am	Assignments—QC
	Practical session—QC	10:15–12:00 pm	
Thursday	Sequence alignment	9:00–11:15 am	Assignments—Sequence alignment and assembly
	Assembly	11:30–1:00 pm	
Friday	Introduction to Git/GitHub	9:00–10:00 am	Assignments—Introduction to the Galaxy—Genomics
	Introduction to the Galaxy	10:15–11:15 am	

Morning sessions covered theoretical concepts, while we dedicated the afternoon sessions to practical sessions and assignments.

Training included two daily sessions, a morning lecture, and an afternoon practical. Participants' feedback from the Symposiums pointed to the need to prepare and share materials early and provide guidance on technical aspects of the program, such as the installation of tools. Before the training, the instructors uploaded the materials to the Canvas Learning Platform (https://www.canvas.net/). The platform allowed sharing of exercises, learning material, and a discussion platform with other course participants. We set aside a day before the main workshop program to assist the participants in setting up workshop material such as GitBash, Ubuntu virtual machine, or a Windows Subsystem for Linux (WSL). We also dedicated an hour before the workshops to answer questions and address any difficulties the participants may have faced the previous day.

**Figure 4:**
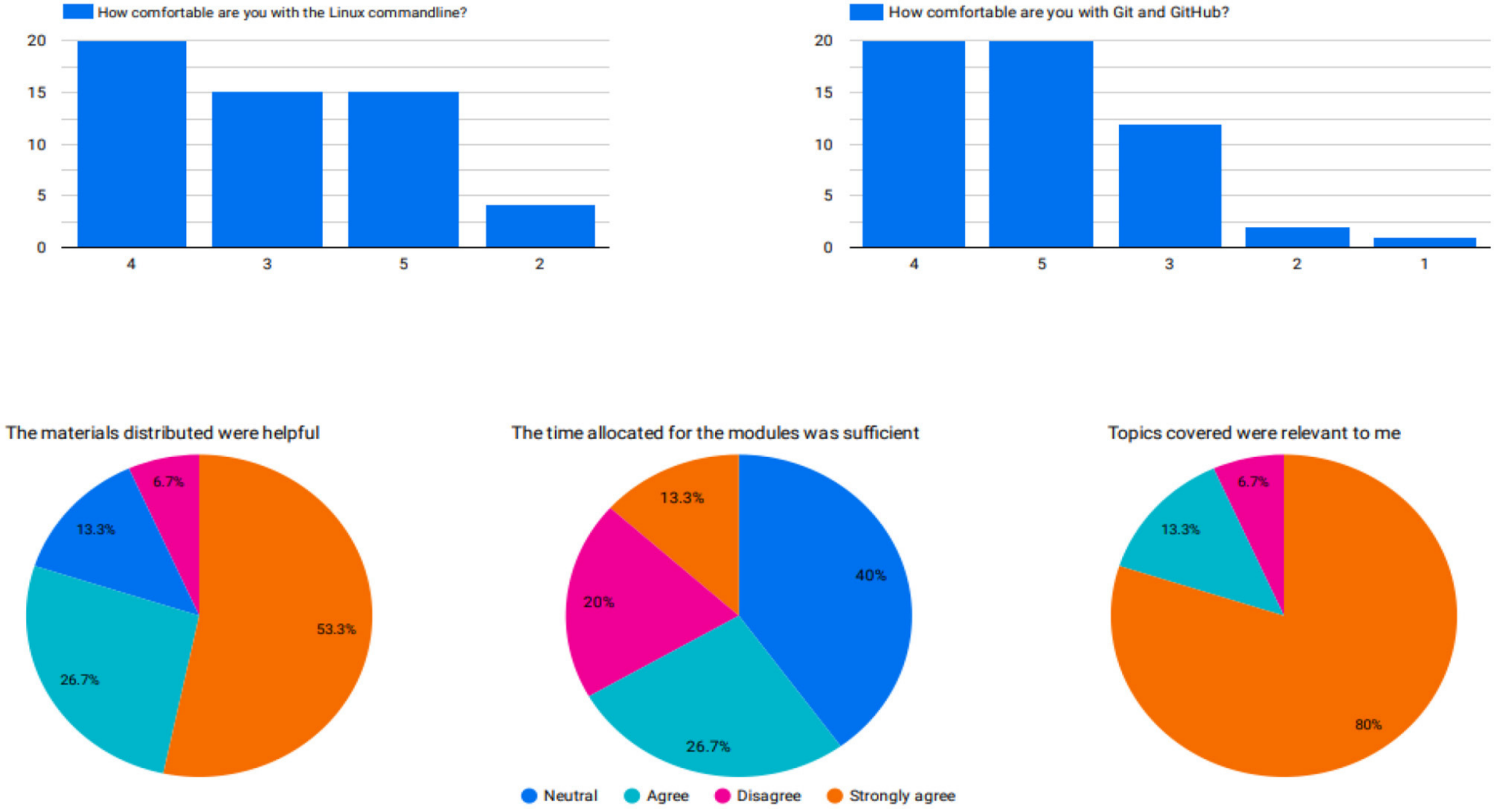
**(Top)** A pre-workshop survey response to assess participants' knowledge of key topics in bioinformatics. **(Bottom)** The post-workshop survey response indicates the satisfaction of participants on selected bioinformatics topics.

After the training, we administered a post-workshop survey to obtain feedback on the sessions. The participants greatly appreciated the programming and practical sessions in the afternoon and requested advanced training workshops. Participants also greatly appreciated training on platforms such as Galaxy since they were not aware of the platform's capabilities. Most participants agreed that the topics covered were relevant to them, the content was well-organized and easy to follow, and the distributed materials were helpful. The time allocated to the sessions was the only challenge for the participants. Although some participants had Internet connectivity problems, 93.3% completed the training. At the end of the training, most trainees were comfortable with the less technical modules, namely sequencing technologies, and familiarization of different data file formats, compared to the more technical ones, namely the Linux command line and quality control assessment (Figure 4).

### Hack: mini-projects

This next phase brought together participants to apply skills acquired in the training phase to collaborative projects. We tasked registered participants for this phase to replicate methodologies and reproduce results obtained in selected papers of different research interests. Participants were assigned mini-projects listed in Table 3 to answer research questions using published data. We designed the mini-projects to demonstrate the need for open and reproducible research while imparting technical skills in genomic data analysis, collaboration, and teamwork. We selected published projects whose data met the FAIR principles and reproducible methods. Each of the five projects—plant genomics, viral research, metagenomics studies, open science in Africa, and research data management handbook—had 5 participants. We assigned a mentor and expert from various institutions to each group.

**Table 3:** Mini project topics, number of participants, and mentors.

**Theme of mini-project**	**Title of the manuscript**	**Members**	**Mentors**
Open science in Africa	Open Science in Kenya: Where are we? (Mwangi et al., 2021)	4	1
Metagenomics	Profiling of RNA viruses in biting butterflies (Ceratopogonidae) and related Diptera from Kenya using metagenomics and metabarcoding analysis (Langat et al., 2021).	6	1
Plant genomics	The draft genomes of five agriculturally important African orphan crops (Chang et al., 2019).	5	2
Viral mini-project	Phylogenetic analysis of the 2020 West Nile Virus (WNV) outbreak in Andalusia (Spain) (Casimiro-Soriguer et al., 2021).	7	2
Open science research data management handbook.	Conceptualized by the BOSS organizing team	3	2

We used GitHub to manage the mini-projects from applications, creating issues for each project and assigning tasks to the participants. Of 31 applicants, we selected 25 based on their experiences and motivation; the selected participants came from Kenya, Tanzania, Uganda, and Ghana. The groups selected a project leader, read their respective manuscripts, prepared a presentation of their approach, and virtually presented it to all participants. The groups met their mentors weekly. The participants then extracted data from the databases in the first week. In the following 2 weeks, groups reproduced the analysis and made a final presentation.

For plant, viral, and metagenomic projects, participants faced challenges around the absence of scripts, sketchy details to reproduce the steps, difficulty coming up with custom scripts for analysis given the timeline, and difficulty using the high-performance cluster. The mentioned challenges significantly affected the completion of mini-projects. The groups working on the Open Science in Africa project completed the project on time. Despite the minimal data science skills of the project participants, they managed to pick up from the already existing code and reproduce results for the status of open science in Africa and interpret it. The last project on the research data management did not start as it did not garner a sufficient response from the participants.

A final presentation was made virtually for each project after 3 weeks, where the participants communicated their progress, challenges, and experience of the mini-projects, with two of the groups presenting at the BOSS conference.

### Sustain: train the trainer

The “Sustain” phase aimed to increase the capacity of trainers in the region through instructor training. We implemented the phase through instructor training by partnering with Carpentries (Teal et al., 2015), which specializes in training subject experts with experience in coding and life sciences to be instructors. The Carpentries instructor training usually involves two phases: instructor training and checkout to become certified. Twenty-five members were selected to join the Carpentries training. Fifteen participants successfully registered for the training sessions: Four did not participate (poor internet connection), Eight completed the training, and six were certified. They gained skills on how people learn, how to provide feedback, teach, and live coding skills, and finally taught in a demo session for their checkout.

### BOSS conference

The Bioinformatics and Open Science Skills (BOSS) virtual conference was the final part of the series of events in the study. The 4-day event themed “Bioinformatics and Open Science Empowerment in an Era of Genomics” brought together researchers and students in bioinformatics, life science, and other fields. Participants joined to present their work, network, and learn from invited speakers on genomics, bioinformatics, open science, careers, and community building. To implement this phase, we formed a 9-member committee to organize the BOSS conference. Planning involved:

- Program design. The committee came up with the conference topics: genomics, open science, research data management, science communication, open science career opportunities, and community development. See the detailed program on the conference website: https://bosscon2022.bhki.org/ and Table 4.
- Speaker selection and communication. One month before the conference, the committee invited experts to give keynotes and talks based on the topics.
- Conference hosting. The committee deliberated over the conference platform needed to accommodate many participants, allow interaction among participants, be easily manageable, and have a low bandwidth requirement. They considered multiple options, including Zoom, BigBlueButton, Aiirmeet, Streamyard, and Eventee, each with strengths and weaknesses. They selected Zoom Events, which is familiar and met the set requirements.
- Conference advertising and registration. The committee used BHKi mailing lists and social networks, primarily Twitter, to raise awareness of the conference and to call for abstract submissions from researchers and students who wanted to present their projects and register for the conference. Free registration through the Zoom Events platform was open for 3 weeks.

**Table 4:** A detailed 4-day BOSS conference program.

**Day 1: open science**	**Day 2: one health, plant genomics, and reproducibility**
Opening remarks (Introduction to the conference)	Keynote: pathogen genomics
Introduction to BHKi and OpenScienceKE	QandA One Health
Icebreaker 1: participants' perspectives of OpenScienceKE and RDM	Plant genomics keynote
Keynote 1: research data management	Q&A plant genomics
Empowered for action: making open science practical	Efforts to identify and combat antimicrobial resistance in Uganda: a systematic review
AfricaRXiv	Reproducibility in the presentation of research projects
Discussion and Q&A	Open science in Africa project presentation
Break	Break/Q&A
GitLab pages for presentation and portfolios	Networking hour: self-assigned breakout rooms
Networking hour: self-assigned breakout rooms	
**Day 3: unconference**	Day 4: genomics and reproducibility
Careers in the community	Welcome and housekeeping
Science communication presentation 1	Keynote: environmental genomics
Alternative career paths within open science	Q&A genomics
Science communication presentation 2	Reproducibility in research: presentation
Break	Open science in Africa project
TCC Africa and its partners	Break
Working and studying outside Kenya: experiences and opportunities	Leveraging cloud genomics
	Large-scale computational regulatory genomics
	Closing remarks

**Figure 5:**
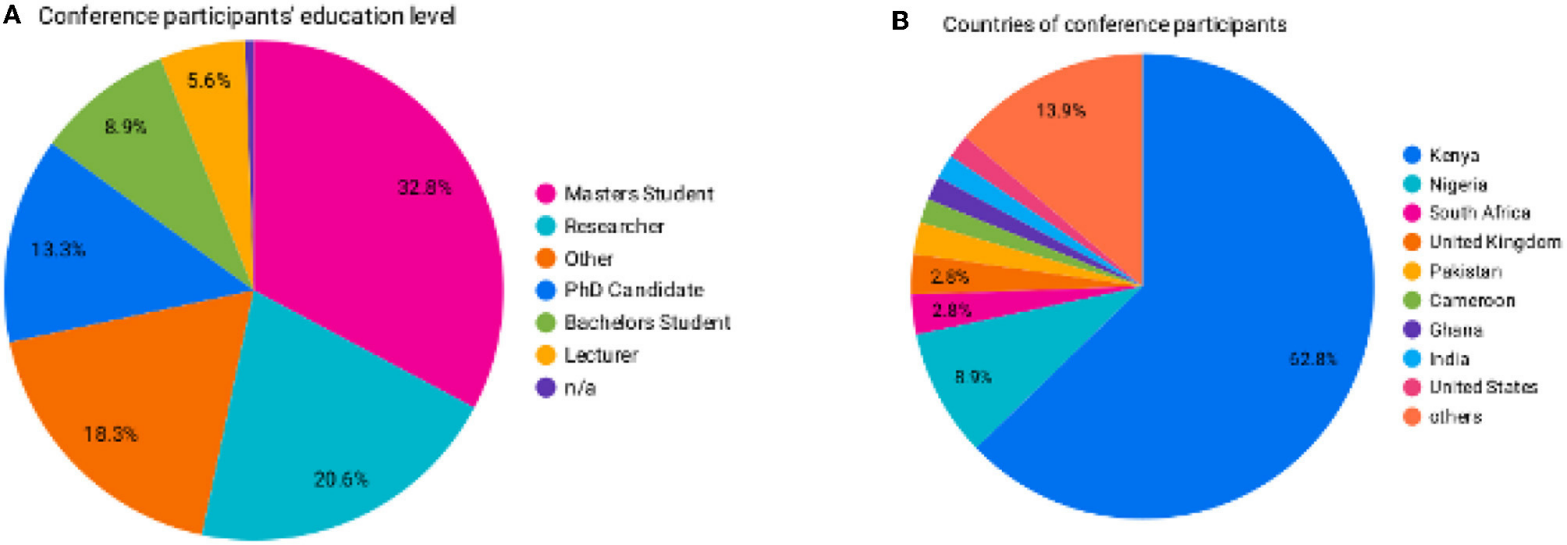
**(A)** The education level of the participants who attended the conference. **(B)** The countries of origin of conference participants.

A total of 207 participants registered: over 60% were from Kenya, and the rest were from Nigeria, Ghana, the United States, South Africa, Cameroon, the United Kingdom, India, and Bangladesh (Figure 5B). The education level of the registrants is shown in Figure 5A.

We had 100 participants attending the conference, with the numbers fluctuating throughout, with a minimum of 50 at any given session. The audience was engaged during all sessions using icebreakers, questions, and polls. The lobby attendance feature of the Zoom Events platform proved advantageous, as participants did not need to log in to Zoom and could follow the proceedings from their browsers. The post-conference survey indicated that the conference met expectations, with 35 participants rating the conference 5, 40 at 4, and 3 at 3.

### Collaborate and community

**Figure 6:**
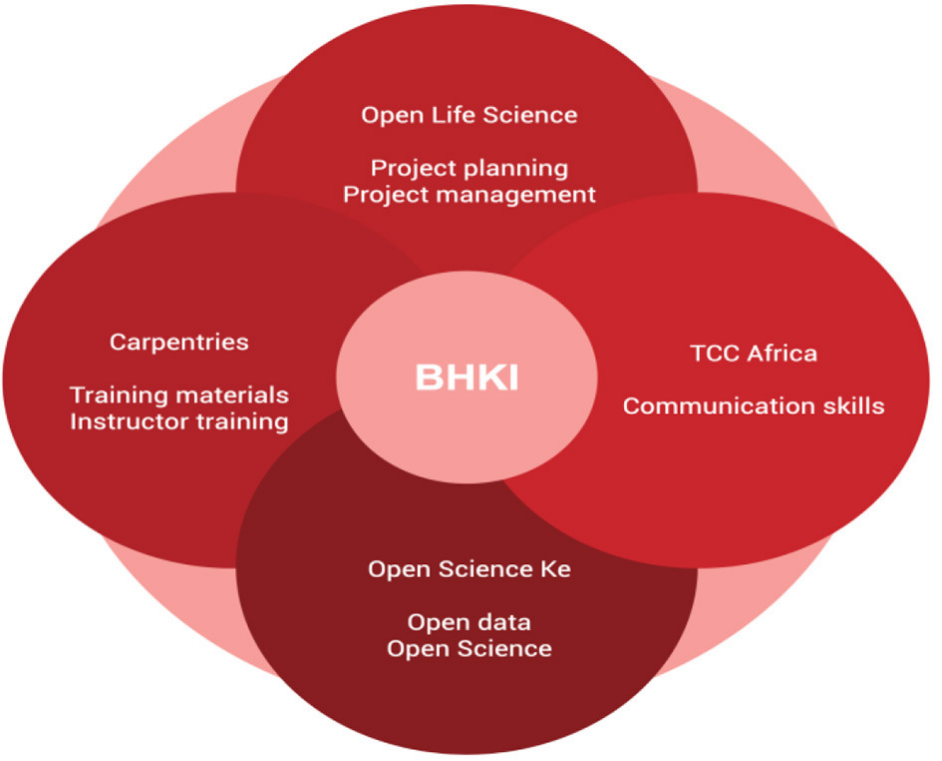
The Grassroot Open Science and Bioinformatics communities collaborated during the events of the Bioinformatics and Open Science Skills.

The African proverb “If you want to go fast, go alone; if you want to go far, go together” aptly captured the motivation behind the “collaborate and community” phase of our events. The impact of our work to sensitize and build capacity in bioinformatics and open science leveraged the power of collaborations. Collaboration and community are the key pillars of the model we used. This phase involves the engagement of the organizations and individuals to put together resources available to the different parties to achieve our shared goal, eliminating the need to reinvent the wheel. Each phase of the model relied on the collaboration of communities. We benefited from communities and organizations such as Human Heredity and Health Bioinformatics network (H3AbioNet), the Carpentries, Training Center in Communication-Africa, Open Life Science (OLS), and individual experts within our networks, as illustrated in the model below (Figure 6).

## Discussion

We have applied the sensitize-train-hack-collaborate model in virtual events to sensitize and empower students and researchers in bioinformatics and open science skills. Though previously developed for in-person events (Mwangi et al., 2021), we demonstrate it is possible to adopt it for virtual events. However, some challenges exist, especially for the hack phase, which works best in person. We also demonstrate the importance of blending open science and bioinformatics awareness and training activities for reproducible and collaborative research. The Global North has successfully defined and adopted bioinformatics and open science practices. The recent pandemic demonstrated the power of having trained personnel with solid skills in bioinformatics and open science as researchers worldwide came together to tackle COVID-19 disease, quickly developing a vaccine (Ball, 2020). Adopting open science and increasing collaboration improved the quality and quantity of research output. Aaron Kessler describes the need to define open science, those who benefit from it, and how its adoption or lack thereof constrains its impact (Kessler et al., 2021). The BOSS events contextualized Open science (Chan et al., 2019); we tailored the activities to a resource-constrained community by designing content, training approach, and supporting participants to facilitate their attendance. The Bioinformatics workshops equipped participants with new tools and techniques, which are rapidly evolving and often take time to diffuse to the global South. We note that early career researchers must be aware of the core competencies they need to generate sound research output, where they can find resources to learn and opportunities to apply their skills. Open science and bioinformatics research differ in the Global North and South, mainly due to the availability of resources and funding. With events being designed and implemented by African researchers and students, we avoid “Trickle-down” science, which does not provide region-specific solutions (Reidpath and Allotey, 2019).

By empowering the participant with pedagogy skills through Carpentries instructor training, we increased the pool of well-trained and skilled research workforce with knowledge of the methods to solve challenges in the region and empower others, thus sustaining the impact of the events.

Creating awareness on the need and benefits of a practice and highlighting how it benefits a particular community increases its adoption. Through the FAIR symposium, participants were sensitized on open science practices. Majority of the participants were from Africa, a testament that researchers in the Global South are interested in and embracing open science and need more information on the benefits and demerits (McKiernan et al., 2016), and what these practices look like in the region. Indeed, we noted that the most impactful sessions were presentations on mapping open science progression in Kenya and Africa, which showed that researchers were interested in those who have already embraced these practices. The presentation on the benefits of open science and how more researchers are adopting these practices helped to demystify some of the misconceptions (Kuchma, 2021), which have slowed the adoption of open science. The eagerness to hear and learn more about open policies by a students would enable them to drive changes where they work and impact decisions made by their departments and advisors (Kathawalla et al., 2021), and enable them to collaborate with other researchers, including from the global North (Allen and Mehler, 2019). The session on publishing in low-cost access journals also garnered much interest, especially from the students in the audience. In some postgraduate programs in Kenya, students must publish in journals as part of the degree requirements (Mwangi et al., 2021). However, they sometimes even lack the funds to conduct their research, let alone pay the article processing fees charged by publishers. To ease this burden of publishing, most of the publishers offer waivers to authors from low- and middle-income countries (Mwangi et al., 2021).

The drive to increase the adoption of open science and current bioinformatics techniques in the global North requires empowering them with the skills and tools. For a long time, helicopter science and extractive research has been the norm, where African researchers are only involved in sample collection, with analysis and publication being driven from the global North. However, training activities such as the BOSS workshops, are changing the landscape, generating a pool of trained students and researcher who can adopt open science and bioinformatics in their research. The BOSS workshop audience consisted mainly of undergraduate, and master's students interested in learning new skills and applying them to their work. Undergraduate students have general knowledge, not techniques they can use for analysis therefore hands-on training is crucial to enable them to understand the theoretical concepts they learn. Masters' students are similarly exposed to theoretical content during the first parts of their program and must learn many techniques as they work on their projects (Brazas et al., 2017). Therefore, they are keen to attend such workshops to learn the skills required for their projects. Although teaching is long, broad, and theoretical, workshops delivers skills in relatively short, practical, and focused courses (Schneider et al., 2010). The BOSS workshops applied practical, experiential and project based learning to facilitate greater skills retention, thus empowering the participants to apply the skill in their research (Emery and Morgan, 2017).

After skill acquisition, there needs to be a practical application of these skills where a participant combines knowledge learned and creativity to produce an outcome, thus enhancing skills retention and collaboration (Ahmed et al., 2019).

The BOSS hack phase employed the use of hackathons and collaborative mini-projects. However, we noted challenges in delivering a hackathon virtually, with only one project out of the four selected being completed within the 3 weeks. The participants had different levels of skill and motivation, which introduced a disconnect in communication (Herrington et al., 2003). They also faced multiple challenges in the initial steps, which proved to be demoralizing and thus affected performance. This phase of the model may have better success through in-person participation, as previously observed (Mwangi et al., 2021). Hackathon and training models developed for resource-constrained regions, should address possible challenges (Jjingo et al., 2022), including offering incentives to participate, such as certification, and support for logistics, such as bandwidth.

Increasing the pool of trainers with pedagogy skills sustains the skills acquisition, and adoption of such practices. Through Carpentries instructor training, the BOSS events enables them to pass on open science practices and tools to new researchers to practice open, reproducible, and collaborative research. There is a need to shift to provide more point-of-need training to equip those with the skills with teaching skills to competently train and assess learners (Attwood et al., 2019; McGrath et al., 2019). Organizations such as Carpentries, European Molecular Biology Laboratory-European Bioinformatics Institute (EMBL-EBI) (www.ebi.ac.uk/training/train-trainer), ELIXIR-EXCELERATE (Morgan et al., 2017) have developed “Train the Trainer” (TtT) programs to teach local members and outside their regions, which we leveraged in this study. Training instructors from a pool of already qualified or skilled individuals enable the sustainability of capacity-building programs (Yarber et al., 2015; McGrath et al., 2019).

Conferences present an opportunity for mentorship, collaboration, brainstorming, networking, the connection of early career researchers with established ones, professional development, and an opportunity for co-learning and community building (Lortie, 2020). However, the cost of travel, visa issues, and conference registration costs often limit the participation of researchers from the global South. Therefore, the shift to virtual conferences has increased the diversity of attendees by eliminating some of the barriers of in-person conferences. The BOSS conference had up to 207 registrants from multiple countries and education level, thus enhancing knowledge spillovers (Jaffe et al., 1993). The online nature of the conference also allowed the use of minimal funds to conduct the conference. The audience was also quite diverse from new demographics, allowing participants to connect across borders and disciplines, noting that research and science are inherently transnational cross-border activities. Virtual conferences allow inclusive, accessible, and equitable meetings (Sarabipour, 2020). However, some challenges driven by systemic inequalities persisted, reducing the participation of under-represented communities (Olzmann, 2020). Providing funding for Internet, childcare, and coworking spaces for the BOSS conference improved accessibility and participation. Funding events such as BOSS allowed the elimination of registration costs, which invited more people to participate. Offering incentives such as Internet support, childcare support, and prizes to those attending events also increased participation. Providing these resources increased the participation of students and researchers without funding for conference attendance.

### Lessons and recommendations

Despite the challenges, the BOSS events were a great success. We reached a large group of participants with the skills and tools in open science and bioinformatics. Virtual events are low-cost solutions to increasing diversity, representation, and participation in training and conferences. We learned the following lessons from the organization and implementation of virtual events that could benefit other communities interested in hosting events using a similar model.

- Collaborate: we can increase the impact and reach of grassroots communities when they collaborate to leverage time, funds, and expertise.
- Localize and contextualize the events: It is essential to understand the challenges and needs of the community when choosing the content, approach, and technology for the events. Use platforms requiring little training and great features to engage an audience and ensure maximum participation.
- Facilitate participation: Increasing diversity is not a matter of reach but removing barriers to participation. Where resources are available, offer funding to support the internet, childcare, and equipment.
- Continuous evaluation: for a series of events, closely monitor and evaluate the approach, the platforms, the needs, and the impact of the events to facilitate continuous improvements.
- Design for Event Networking: Symposia and conferences are platforms for networking, sharing ideas, and serendipitous encounters that lead to collaborations. It is crucial to design the sessions to include networking hours, chat features, and discussion among the participants.
- Practice what you preach: It was essential to embrace openness by design for open science in the bioinformatics series of events. We shared all materials with an open license and used open-source tools.
- Offer incentives, including certification and prizes, and invite more local speakers in an unconference theme style that participants can relate to, encouraging participation and engagement.

## Conclusions

Open science in bioinformatics facilitates reproducibility and collaboration, a skill greatly needed in a heavily computational field. Therefore, it is vital to impart open science skills to bioinformatics students and researchers, especially when training in open science tools accompanies bioinformatics training. Through the Open Science Skills Bioinformatics events, we applied a modified OpenScienceKE framework, which proved to be a suitable model to introduce and train open science and bioinformatics skills. We show that sensitizing and empowering researchers with the skills works best when the participants apply the skills to real-world problems: project-based learning. Furthermore, we have demonstrated how to implement virtual events in resource-constrained settings by providing Internet and equipment support to participants, thus improving accessibility and diversity. Challenges exist, especially with virtual events, such as poor connectivity and high dropout rates. Sharing the recordings after the sessions ensures that participants can catch up on the content, thus leaving no one behind. Virtual events are here to stay, even when the pandemic is over. Therefore, we must understand the opportunities and challenges and tailor the approaches and tools in different contexts.

## Data availability statement

The datasets generated for this study can be found in the BHKi GitHub repository: Workshops data—https://github.com/bioinformatics-hub-ke/Boss-workshops while resources for the BOSS; Miniprojects data—https://github.com/bioinformatics-hub-ke/BOSS-miniprojects.

## Author contributions

PK wrote the initial draft of the manuscript while being supported and guided by CK, and the rest of the authors made significant contributions to the manuscript. All authors participated in sourcing funding, organizing, and facilitating various activities described in the paper.
